# Erythromelalgia: An Uncommon Presentation Precipitated by Aspirin Withdrawal

**DOI:** 10.1155/2012/616125

**Published:** 2012-07-16

**Authors:** Fatima Khalid, Syed Hassan, Sophia Qureshi, Waqas Qureshi, Syed Amer

**Affiliations:** ^1^Department of Internal Medicine, Henry Ford Hospital, 2799 W Grand Blvd., Detroit, MI 48202, USA; ^2^Department of Internal Medicine, Rawalpindi Medical College, Rawalpindi 46000, Pakistan

## Abstract

Erythromelalgia is a rare disorder frequently associated with myeloproliferative disorders. We describe a case of elderly patient diagnosed with myeloproliferative disorder in remission. The patient was on aspirin for secondary prevention of stroke and was taken off aspirin and developed erythromelalgia within two weeks of withdrawal of aspirin. After restarting aspirin, patient's symptoms improved within 2 weeks.

## 1. Introduction

Erythromelalgia is a rare condition of unclear etiology characterized by redness, warmth, and severe burning of lower extremities. It can frequently go unrecognized because it may mimic other dermatological conditions [1]. It is more common in women than in men [2]. It most frequently affects the lower extremities followed by upper extremities [3]. Rarely, it can even affect the face and the ears [4]. Symptoms are episodic and can result in severe debility because of intolerable pain. Patients often function normally in between the episodes. 

The diagnostic criteria for erythromelalgia suggested by Thompson et al. [5] are as follows: (1) burning pain in the extremities, (2) pain decreased by cooling, (3) pain increased by warming, (4) erythema of the affected skin region, and (5) increase in temperature of the affected skin. Erythromelalgia is classified into two types, primary and secondary. Primary is further divided into idiopathic, familial, and sporadic. Secondary is associated with essential thrombocythemia [6], polycythemia [6], myelodysplastic syndrome [7], pernicious anemia, acute diabetic nephropathy [8, 9], multiple sclerosis [10], systemic lupus erythematosus [11], HIV [12], or use of medications like verapamil, nifedipine [13], and bromocriptine [14].

It is postulated that abnormal platelet aggregation might be a causative factor of erythromelalgia and some of the types of erythromelalgias have responded to aspiring [15]. Although, there has not been a case where withdrawal of aspirin has led to development of erythromelalgia.

## 2. Case Report

**Figure 1:**
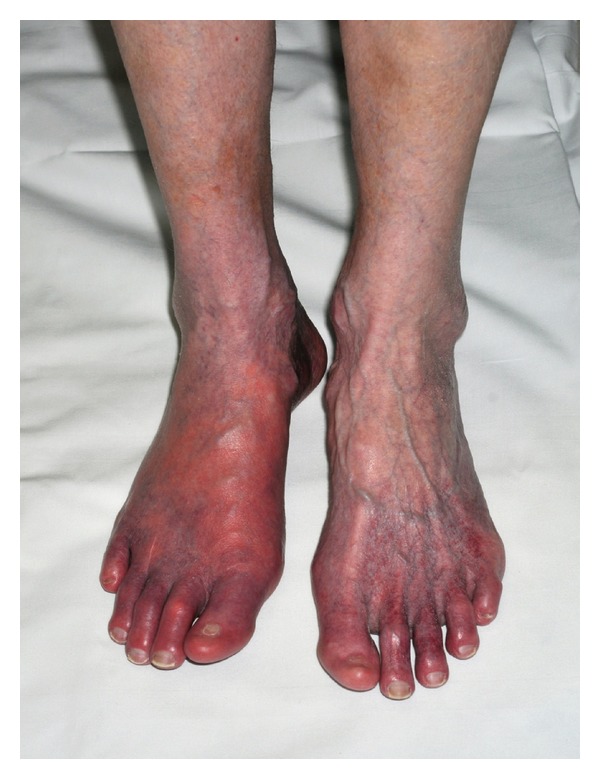
Before initiation of aspirin.

**Figure 2:**
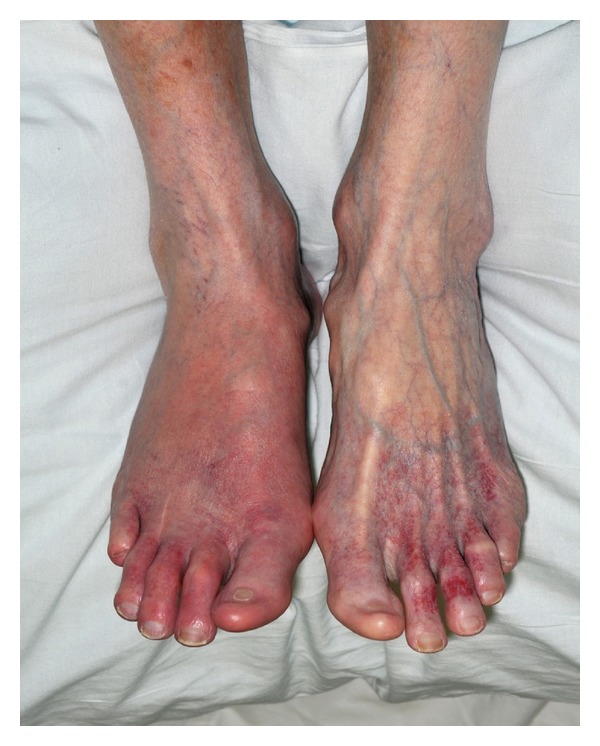
24 hours after treatment with aspirin.

We present a case of a 64-year-old caucasian female who presented to the emergency department with bilateral foot pain, erythema, and redness more prominent in the right foot (Figure 1). The pain was excruciating, 10/10, burning in quality and worsened with ambulation as well as being placed in a dependent position. Her medical history was significant for hypertension, myelodysplastic syndrome disorder in remission, and stroke two years ago. She was placed on aspirin after that for secondary prevention of stroke. Two weeks prior to this presentation, she was found to have right iliac artery thrombosis and anticoagulation with coumadin was initiated with discontinuation of the aspirin. One week prior to this presentation, she presented to another hospital where she was treated for provisional diagnosis of cellulitis and patient was sent home on antibiotics, although she returned, since the symptoms were not improving. There was no history of trauma. Other medications included gabapentin, omeprazole, and atenolol. On examination, she was hemodynamically stable and afebrile, distal pulses were intact, and there was blanchable erythema in the bilateral lower extremities. The erythema worsened on changing the position of the legs from supine to dependent position. One peculiar thing was that she complained of more severe pain and redness when she wore socks or putting the blanket on her legs and felt some relief with a cooling fan by her legs or pouring cold water down her legs. There were no sensory deficits to light touch and pin prick. Monofilament testing was normal. Laboratory studies showed normal white blood cell and platelet count with an elevated international normalized ratio (INR). Initial differential diagnosis including cellulitis and arterial or venous insufficiency were systematically excluded. A presumptive diagnosis of erythromelalgia was made taking into account that she fulfills all of the Thompsons's criteria. Aspirin 325 mg was initiated. The patient showed significant improvement in her symptoms within 24 hours of initiation of aspirin therapy (Figure 2), her pain and redness came down significantly from 10/10 to 3/10, both upon ambulation and with rise in room temperature, she was able to walk pain-free for the first time in 2 weeks. However, the erythema and redness persisted for some time. After a followup of two weeks, her symptoms completely resolved and she was advised to continue taking the aspirin.

## 3. Discussion

Symptoms of erythromelalgia can be devastating for the patients. It is associated with significant morbidity [16]. It is a complex disease and various theories have been suggested to explain its pathogenesis. Some authors have suggested that patients with erythromelalgia have a predominantly small-fiber neuropathy [17]. Others suggest that the redness and warmer temperature of the affected limbs are due to increased microvascular arteriovenous shunt flow [18, 19]. 

Several treatment modalities have been tried for it. They include aspirin, indomethacin, beta-blockers, calcium channel antagonists, misoprostol, diltiazem, tricyclic antidepressants, gabapentin, and serotonin reuptake inhibitors. In resistant cases more aggressive forms of treatment have been tried. These therapeutic options include modalities such as intravenous lidocaine, epidural anaesthesia, intrathecal opiates, and sympathetic ganglion blockade [20]. Sodium channel blockers like ranolazine and mexiletine have shown promising response in patients with primary erythromelalgia [21].

Prior case descriptions of erythromelalgia describe this entity in the setting of significantly elevated platelet counts which decline into a normal range after initiation of aspirin [22–24]. Our case is unique in that the platelet count was normal at the time of presentation, likely as a result of patient being on anagrelide. This emphasizes that erythromelalgia can still develop in the presence of normal platelet counts in patients with myeloproliferative disorder.

Another unusual feature is that the symptoms were precipitated by withdrawal of aspirin in a patient who did not have a known diagnosis of erythromelalgia. This implies the presence of prostaglandin-mediated disturbance in the pathogenesis of this disease.

Also, gabapentin which was considered in a previous case report as the treatment modality for erythromelalgia did not seem to affect the symptoms in this patient [8, 25, 26]. It might be possible that the pathogenesis of this disease might be due to various initial triggers or target structures ranging from vessels, platelets to small nerve fibers.

Further research is needed for a more complete understanding of the pathophysiology of erythromelalgia and this might subsequently help to formulate definite guidelines on its management.
